# Development and Validation of a Neural Network Model for Predicting Atrial Fibrillation and Detecting Silent Arrhythmias in Patients with Chronic Obstructive Pulmonary Disease Based on Echocardiography Data

**DOI:** 10.3390/diseases14060206

**Published:** 2026-06-09

**Authors:** Stanislav Kotlyarov, Alexander Lyubavin

**Affiliations:** 1 Department of Nursing, Ryazan State Medical University, 390026 Ryazan, Russia; alexlubavin48@gmail.com; 2 Lipetsk City Hospital No. 4, 398006 Lipetsk, Russia

**Keywords:** atrial fibrillation, chronic obstructive pulmonary disease, neural networks, myocardial remodeling, artificial intelligence, echocardiography, prognosis

## Abstract

Background: Atrial fibrillation (AF) is a common arrhythmia with a high incidence, and patients with chronic obstructive pulmonary disease (COPD) are at particularly high risk. However, there are currently no tools available for early risk stratification of AF in this population. Objectives: To develop and validate a neural network diagnostic model based on transthoracic echocardiography to address two clinical challenges in patients with COPD: risk stratification for AF; and detection of occult supraventricular arrhythmias (including “micro-AF”) based on 24 h ECG monitoring data. Methods: The study consisted of three consecutive stages: development of a neural network (NN) based on transthoracic echocardiography (TTE) parameters, validation of the model’s predictive ability in patients (*n* = 311, including 99 with COPD), and assessment of the ability to detect occult atrial arrhythmias (*n*=207) in patients with COPD. The model architecture consists of a fully connected multilayer perceptron (MLP) with 13 inputs, 4 hidden layers of 130 neurons each, and 2 output neurons. Training was performed on 684 TTE scans (292 without AF, 392 with AF). The echocardiographic parameters were validated on an independent test set (*n* = 100). Statistical analysis included pairwise and multiple comparisons, logistic regression analysis, and ROC analysis with assessment of the area under the ROC curve (AUC). The median follow-up period for study participants was 18 months. Results: The neural network demonstrated high classification metrics for AF on the test set (AUC = 0.80). A threshold value of the first output layer neuron > 0.75 allowed for the identification of a high-risk subgroup, in which the incidence of AF in patients with COPD was 14.8% versus 0% in the low-risk subgroup (*p* = 0.0073). Logistic regression models of the relationship between AF development and the neural network output value were statistically significant in both patients with COPD and patients without COPD (*p* < 0.0001). In patients with COPD without a history of AF, the neural network identified a high-risk group. In this group, 24 h ECG monitoring more frequently recorded episodes of AF, group supraventricular extrasystoles, and the combined endpoint (AF + GSE) compared to the low-risk group (55.32% vs. 17.5%; *p* < 0.0001). The area under the ROC curve for detecting latent AF in patients with sinus rhythm based on the neural network prediction was 0.93. Conclusions: The developed neural network model, which integrates a set of TTE parameters into a single quantitative measure of the severity of myocardial remodeling, is an effective tool for risk stratification for AF. The model may help identify COPD patients who could benefit from intensified rhythm monitoring; however, external validation is required before clinical implementation.

## 1. Introduction

Atrial fibrillation (AF) is a common arrhythmia associated with a fivefold increase in the risk of thromboembolic complications, the development of heart failure, and a reduced quality of life for patients [1,2]. Despite significant advances in understanding the pathophysiology of AF, up to 13% of cases remain undiagnosed, with more than half of these involving patients at moderate or high risk of stroke [3]. This problem is particularly acute in the presence of comorbidities that modify the course of the arrhythmia and worsen the prognosis.

Chronic obstructive pulmonary disease (COPD) is one of the most significant risk factors for the development of AF [4]. According to the results of large epidemiological studies, the presence of COPD increases the likelihood of atrial fibrillation by 1.7–2.0 times [5]. Moreover, the comorbidity of AF and COPD is associated with a more severe course of both diseases. Patients with this combination are more likely to have hypertension, diabetes mellitus, and chronic heart failure. They also exhibit higher 30-day rehospitalization (16.0% vs. 9.0%) and mortality rates compared to those with isolated forms of these diseases [6,7].

AF develops through electrophysiological and structural remodeling of the atrial myocardium. In patients with COPD, myocardial remodeling is biventricular in nature, affecting not only the left but also the right chambers of the heart, which creates an expanded arrhythmogenic substrate [8].

In recent years, there has been active development in the field of applying artificial intelligence and neural network technologies for the diagnosis and prognosis of cardiovascular diseases. Artificial intelligence has already demonstrated its effectiveness in optimizing AF screening by improving patient selection for in-depth examination, as well as in enhancing the diagnostic capabilities of electrocardiography, ensuring accurate detection of arrhythmias [9,10,11]. Neural network models utilizing echocardiography data are also able to assess structural and functional changes in the myocardium that create a substrate for arrhythmogenesis, enabling the identification of patients at high risk of developing AF [12].

Despite existing advances, the available literature lacks specialized algorithms for predicting AF in patients with COPD, who exhibit complex pathophysiological interactions between respiratory dysfunction and myocardial remodeling. Most existing models are designed for the general population and do not account for the specific cardiopulmonary interactions characteristic of COPD. Furthermore, existing approaches are primarily aimed at predicting the risk of developing AF, but not at identifying pre-existing occult (asymptomatic) forms of arrhythmia, including so-called “micro-AF” (grouped supraventricular extrasystoles and brief episodes of supraventricular tachycardia), which may serve as markers of undiagnosed AF. The development of an effective tool for stratifying AF risk in this patient population could help optimize patient management strategies, enable timely initiation of anticoagulant therapy, and reduce the incidence of thromboembolic complications.

This study proposes a neural network diagnostic model based on transthoracic echocardiography, specifically designed for patients with COPD. The novelty of this study lies in the fact that the model is intended to address clinical challenges related to predicting the risk of atrial fibrillation and detecting occult supraventricular arrhythmias in patients with COPD. We hypothesized that a neural network combining standard echocardiographic parameters could quantitatively assess myocardial remodeling and identify COPD patients at high risk of developing AF, as well as be used to detect occult supraventricular arrhythmias based on 24 h ECG monitoring data.

The aim of this study was to develop and validate a neural network diagnostic model based on transthoracic echocardiography to address two clinical challenges in patients with COPD: risk stratification for AF, and detection of occult (silent) supraventricular arrhythmias (including “micro-AF”) based on 24 h ECG monitoring data.

## 2. Materials and Methods

### 2.1. Study Design and Ethical Approval

The study was approved by the Ethics Committee of Ryazan State Medical University (Protocol No. 4 dated 9 October 2023). The study was conducted at the cardiology department of the Lipetsk City Hospital No. 4 “Lipetsk-Med” State Healthcare Institution.

**Figure 1:**
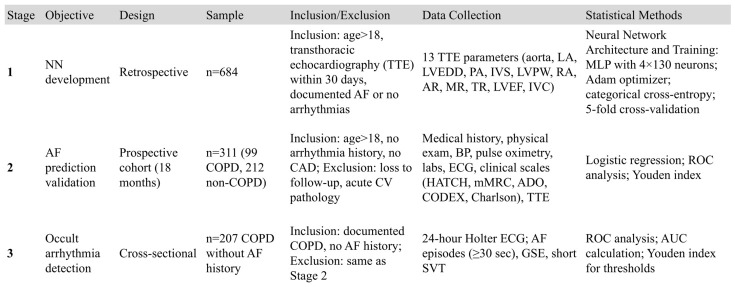
Study Design. Abbreviations: ADO—Age, Dyspnea, Obstruction (COPD prognostic score); AF—atrial fibrillation; AUC—area under the curve; BP—blood pressure; CAD—coronary artery disease; Charlson—Charlson Comorbidity Index; CODEX—Comorbidity, Obstruction, Dyspnea, Exacerbations (COPD severity score); COPD—chronic obstructive pulmonary disease; CV—cardiovascular; ECG—electrocardiogram; FEV_1_—forced expiratory volume in 1 s; GSE—group supraventricular extrasystoles; HATCH—Hypertension, Age, Transient ischemic attack/stroke, Chronic heart failure, COPD (AF risk score); HR—heart rate; IQR—interquartile range; IVC—inferior vena cava; IVS—interventricular septum; LA—left atrium; LVEDD—left ventricular end-diastolic diameter; LVEF—left ventricular ejection fraction; LVPW—left ventricular posterior wall; MLP—multilayer perceptron; mMRC—modified Medical Research Council (dyspnea scale); MR—mitral regurgitation; NN—neural network; PA—pulmonary artery; RA—right atrium; ROC—receiver operating characteristic; SD—standard deviation; SVT—supraventricular tachycardia; TR—tricuspid regurgitation; TTE—transthoracic echocardiography.

The study consisted of three consecutive phases, each conducted on independent cohorts: (1) development of a neural network for the quantitative assessment of myocardial remodeling in AF—based on transthoracic echocardiography (TTE); (2) validation of the neural network’s prognostic ability regarding the development of AF in patients with COPD; (3) assessment of the neural network’s ability to detect asymptomatic atrial arrhythmias in patients with COPD (Figure 1).

### 2.2. Stage 1. Development of a Neural Network Model

#### 2.2.1. Participants

The analysis included clinical data from 684 patients permanently residing in the Lipetsk region who underwent examination at the Lipetsk City Hospital No. 4 between 1 January 2022, and 31 December 2023, and who met the inclusion criteria. This stage was conducted retrospectively based on an analysis of medical records.

Inclusion criteria: age over 18 years; transthoracic echocardiography (TTE) performed no more than 30 days prior to inclusion; for patients without AF—no documented history of arrhythmias (atrial fibrillation/flutter, supraventricular tachycardia, polymorphic supraventricular extrasystoles, ventricular tachycardia); for patients with AF—documented AF based on a 12-lead ECG or 24 h ECG monitoring (episode lasting > 30 s).

#### 2.2.2. Echocardiography

All patients underwent TTE using an Accuvix V10 ultrasound system (Medison Co., Ltd., Seoul, South Korea). The following parameters were assessed:

- In the parasternal long-axis view: ascending aorta diameter (cm), left atrium diameter (cm), left ventricular end-diastolic diameter (cm);
- In the parasternal short-axis view: pulmonary artery diameter (cm), maximum thickness of the anterior and posterior walls of the left ventricle (cm);
- In the apical four- and five-chamber views: transverse dimension of the right atrium (cm), degree of regurgitation at the aortic, mitral, and tricuspid valves (assessed visually using color Doppler mapping and coded as a continuous variable: 0—absent, 1—minimal, 2—moderate, 3—severe); left ventricular ejection fraction (Simpson method, %).

#### 2.2.3. Neural Network Architecture and Training

The model was implemented in Python 3.9 using the TensorFlow library (source code available on GitHub: https://github.com/alexlubavin/af_predict_network (accessed on 4 June 2026)). The architecture is a fully connected multilayer perceptron (MLP) with 13 input neurons (corresponding to 13 echocardiographic parameters), 4 hidden layers with 130 neurons each, and 2 output neurons (classification: AF/non-AF). The network consists of 535 neurons with a total of 52,650 trainable parameters.

The input data were normalized and scaled to values between 0 and 1 using the formula: r = (n − min)/(max − min), where n is the value of the quantitative variable, max is the maximum value of the variable, and min is the minimum value of the variable in the sample. The degree of regurgitation was treated as a continuous variable for correct neural network data processing. The data were then passed for processing as a two-dimensional matrix of 684 vectors of 15 values each (13 values of TTE parameters for the input neurons and 2 values for the output neurons—AF or non-AF).

The sample (*n* = 684) was divided into three independent subsamples while attempting to preserve the original class proportions (292 without AF, 392 with AF):

- Training sample: 500 vectors (200 without AF, 300 with AF);
- Validation set: 84 vectors (28 without AF, 56 with AF);
- Test set: 100 vectors (34 without AF, 66 with AF).

**Figure 2:**
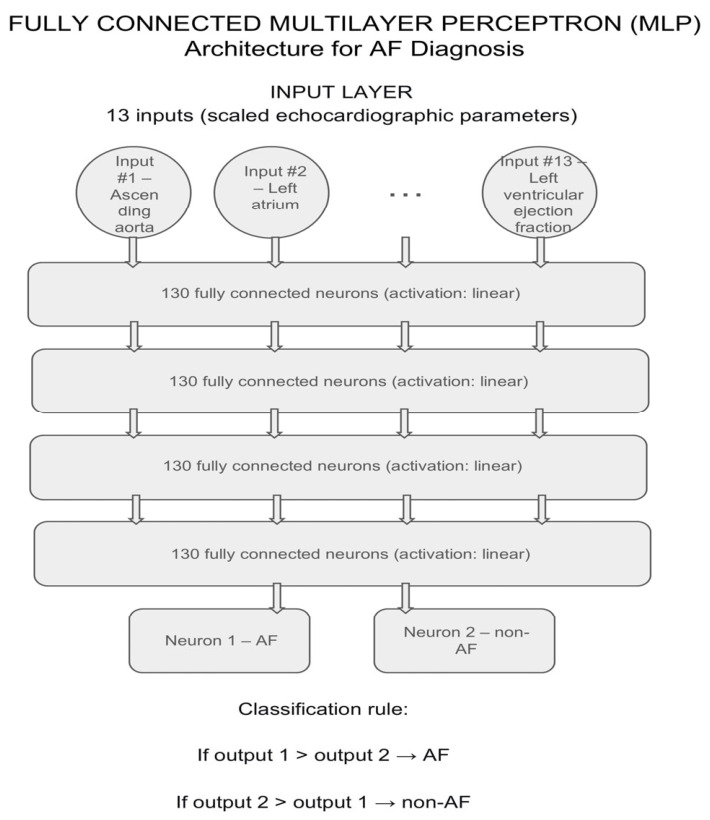
Neural Network Architecture.

Training: backpropagation method with the Adam optimizer. Loss function: categorical cross-entropy. Classification was performed according to the following rule: if the value of the first output neuron exceeds the value of the second, AF is diagnosed; if the value of the second output neuron exceeds the value of the first, the neural network considered the TTE as not characteristic of AF (Figure 2).

Quality evaluation: ROC analysis on balanced datasets and Precision–Recall (PR) analysis were used to evaluate the model’s performance under class imbalance (AF rate <10%). The classification threshold was selected based on the maximum F1 score (the harmonic mean of precision and recall).

Training results on the test set:

- Accuracy = 0.81;
- Precision = 0.87;
- Recall = 0.83;
- AUC ROC = 0.80 (95% CI 0.71–0.87).

To quantitatively assess the severity of myocardial remodeling processes, the study considered the values of the first and second outputs of the NN, and the difference between the output values was calculated. Based on the ROC analysis performed on the test sample for various values of the first NN output, a value of 0.75 for the first NN output was selected as the criterion for a high probability of AF development to divide the sample into groups: when the neural network output was greater than 0.75, the risk of developing AF was considered high; when the neural network output was less than or equal to 0.75, the risk of developing AF was considered low (Supplementary Table S1).

To evaluate the performance of the developed model in comparison with other algorithms, a 5-fold cross-validation was performed on the training set (*n* = 500). Logistic regression yielded an AUC of 0.702 ± 0.111, random forest—0.804 ± 0.031, gradient boosting—0.759 ± 0.054, and the support vector machine method—0.750 ± 0.037. The proposed neural network achieved an AUC of 0.80 ± 0.03, which is comparable to the random forest, but at the same time provides a simple interpretation through a single output value.

### 2.3. Stage 2. Validation of the Predictive Ability of NN in Patients with COPD

#### 2.3.1. Participants and Design

The analysis included 311 patients (of Caucasian descent, permanent residents of the Lipetsk region) who were followed between 11 October 2023, and 10 October 2025. The sample did not overlap with the sample from Stage 1.

Inclusion criteria: age > 18 years, no history of cardiac arrhythmias: atrial fibrillation, atrial flutter, supraventricular tachycardia, polymorphic supraventricular extrasystoles, ventricular tachycardia; no history of angiographically confirmed coronary artery atherosclerosis, previous myocardial infarction, or myocardial revascularization procedures.

Exclusion criteria: inability to follow up (change in residence), development of acute cardiovascular pathology altering the geometry of the heart and valves (myocardial infarction, myocarditis, endocarditis, pericarditis).

All patients underwent medical history collection (including comorbidities), physical examination, blood pressure measurement using the Korotkov method (MT-20 mechanical sphygmomanometer, MediTech, Saint Petersburg, Russia), pulse oximetry (B-Well MED 320, B. Well Swiss, Widnau, Switzerland), standard laboratory tests (complete blood count and urinalysis, biochemistry, lipid profile), and a 12-lead ECG (EK12T-01-RD device, Rostov-on-Don, Russia). The following clinical scales were assessed: HATCH (risk of AF), mMRC (dyspnea), ADO and CODEX (severity of COPD), and the Charlson Comorbidity Index. Echocardiography was performed using the method described above.

#### 2.3.2. Follow-Up and Endpoints

The median follow-up period was 18.0 [14.0; 21.0] months. Data were collected using electronic medical records (the “Quasar” Medical Information System, MedSoft LLC, Lipetsk, Russia), in-person visits, and telephone calls. Endpoints: seeking medical care for AF or reporting AF as a complication or comorbid condition.

### 2.4. Stage 3. Identification of Asymptomatic Arrhythmias in Patients with COPD

The analysis included 207 patients with COPD and no history of AF who met the inclusion criteria for Stage 2, with the additional mandatory requirement of a documented diagnosis of COPD. Recruitment period: 11 October 2025–28 February 2026. The inclusion/exclusion criteria were similar to those of Stage 2; an additional inclusion criterion was documented COPD.

In this study, 24 h ECG monitoring was performed using the “Valenta M 02-05” system (Russia). The following were assessed: the presence of episodes of atrial fibrillation (duration ≥ 30 s); group and multifocal supraventricular extrasystoles; short episodes of supraventricular tachycardia (<30 s)—“micro-AF”; ventricular premature beats (total number, paired, group).

Endpoints: detection of AF during 24 h monitoring; detection of group supraventricular extrasystoles (GSE).

### 2.5. Statistical Analysis

Data were analyzed using MedCalc software (version 22.016, MedCalc Software, Ostend, Belgium). The normality of the distribution was tested using the Shapiro–Wilk test. Quantitative data with a normal distribution are presented as M ± SD, and those with a non-normal distribution as Me [25%; 75%]. To compare two independent groups, we used Student’s *t*-test (for normal distributions) or the Mann–Whitney U-test. For comparing categorical variables, the χ^2^ test or Fisher’s exact test was used. For multiple comparisons, the Bonferroni correction was applied (significance threshold for three groups—*p* < 0.0167; for four groups—*p* < 0.00833). Logistic regression models were constructed to identify factors associated with the development of AF. ROC analysis was performed, calculating the area under the curve (AUC), sensitivity, specificity, and the optimal threshold using the Youden index. Differences were considered statistically significant at *p* < 0.05.

## 3. Results

### 3.1. Predicting the Development of Atrial Fibrillation Using a Neural Network

#### 3.1.1. Characteristics of Study Participants

Since the developed neural network allows for the quantitative assessment of the severity of myocardial remodeling in AF, patients with COPD were selected to validate its predictive ability. In this patient group, remodeling affects both the right and left chambers of the heart, which suggests a higher predictive power of the model. To validate the predictive ability of the developed neural network, an analysis of medical records was conducted for 311 patients aged 19–96 years (mean age 64.27 ± 14.27 years, median age 68.0 years), of whom 148 (47.59%) were women. This sample did not overlap with the sample used to train the AI model. The median follow-up period was 18.0 [14.0; 21.0] months. The main characteristics of the patients are presented in Table 1. During the follow-up period, atrial fibrillation developed in 18 (5.79%) participants; the median time to the first episode of AF was 6.5 [5.25–10.5] months. During the study, 28 (9.0%) participants died; the median time to death was 8.5 [3.75–15.25] months.

Next, the study participants were divided into two groups. The first group consisted of 99 patients with COPD, and the second group consisted of 212 patients without COPD (Table 2).

The group of participants with COPD was older and included fewer women. The groups differed in the number of deaths, with a higher frequency and earlier onset of fatal outcomes in the group of patients without COPD (*p* < 0.05), which is due to the high prevalence of severe non-cardiac pathology.

The study included patients with various forms of COPD (the median COPD stage was 3.0 [3.0–4.0]; 18 participants (18.18%) had stage II COPD, 55 participants (55.56%) had stage III COPD, and 26 participants (26.26%) had stage IV COPD). Smoking duration was 33.43 ± 8.85 years, pack-years index was 33.48 ± 8.96 pack-years, and FEV1 was 54.71 ± 11.21% (Supplemental Table S2).

Participants in both groups did not differ significantly in terms of primary comorbidities, mortality from CVD, and CHF. It is worth noting that the group of patients with COPD did not include patients with hypothyroidism, dilated cardiomyopathy, or aortic valve stenosis. Despite the exclusion from the study of patients with proven IHD, myocarditis, endocarditis, and pericarditis, the study included patients with a number of comorbidities (the Charlson Comorbidity Index was 2.63 ± 1.54) and a moderate risk of developing AF (the risk of developing AF on the HATCH scale was 1.85 ± 1.14). Patients with COPD had a higher risk of developing AF according to the HATCH score and a higher proportion of patients with more than 3 points on this scale (*p* < 0.0001).

#### 3.1.2. Echocardiographic Differences

**Figure 3:**
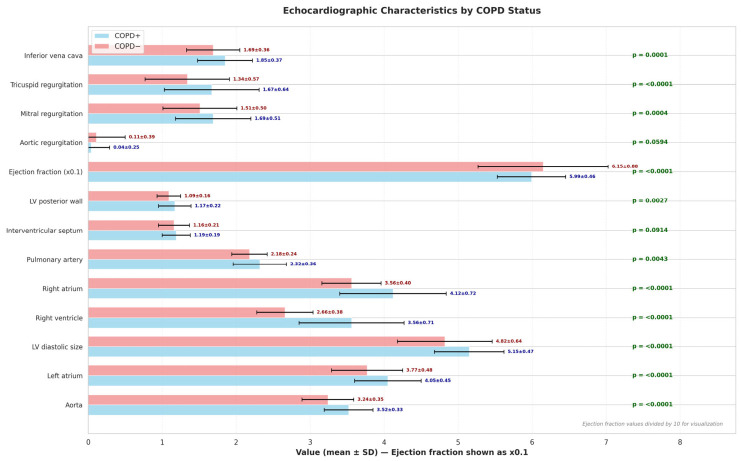
Echocardiographic parameters of the study participants.

**Figure 4:**
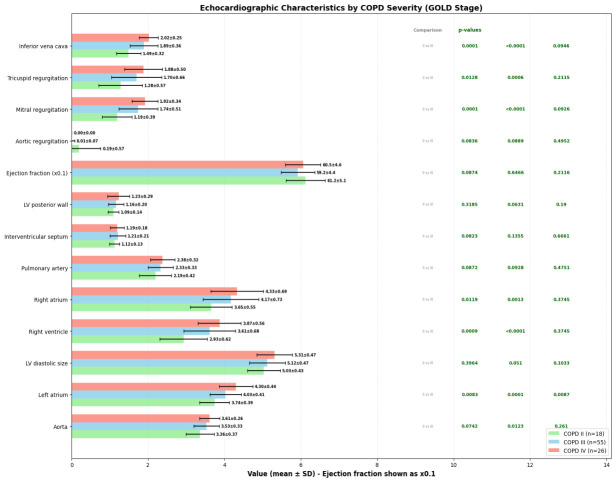
Echocardiographic characteristics by COPD severity.

Patients in both groups differed in a number of echocardiographic parameters. Thus, the group of patients with COPD was characterized by larger dimensions of the aorta, left atrium, left ventricular end-diastolic diameter, right ventricle, right atrium, and inferior vena cava diameter (*p* < 0.0001). In addition, patients with COPD had a more pronounced degree of regurgitation at the mitral and tricuspid valves, and the severity of echocardiographic abnormalities in the COPD group increased in accordance with the stage of COPD (Figure 3 and Figure 4, Supplemental Tables S3 and S4).

#### 3.1.3. Logistic Regression Models

**Figure 5:**
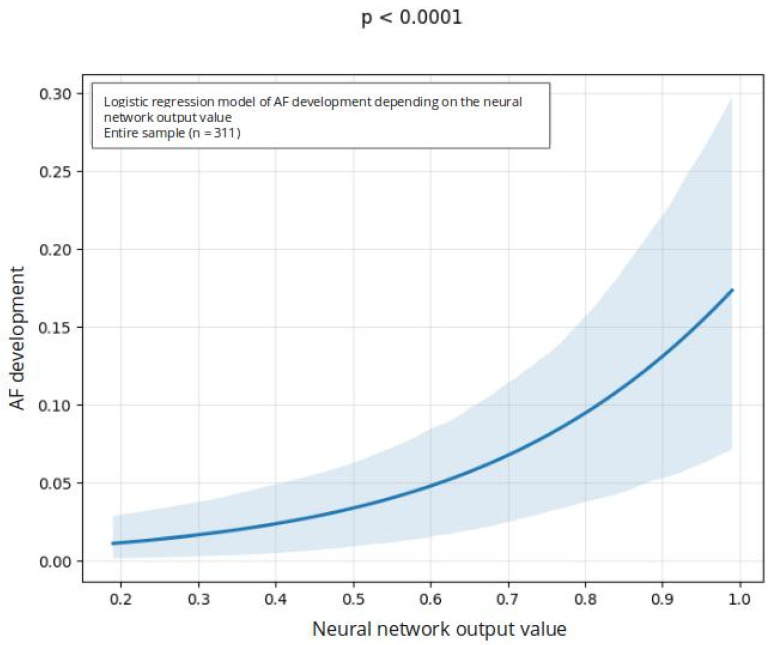
Logistic regression of the dependence of AF development on the neural network output value.

Based on the values obtained at the first output of the neural network, logistic regression models were constructed, reflecting the dependence of AF development on the values obtained at the neural network output. Statistically significant models (*p* < 0.0001) were obtained for the COPD group, the non-COPD group, and the entire sample; the highest coefficient was observed in the group of patients with COPD, indicating the greatest predictive power of the neural network specifically in this group of participants (Table 3, Figure 5).

#### 3.1.4. Analysis of High- and Low-Risk Subgroups in Patients with COPD

Based on the data on the prediction of AF development in the group of participants with COPD and the construction of an error matrix, an analysis of the metrics of the predictive neural network model was performed, taking into account the sample imbalance. The Precision–Recall (PR) analysis identified the best threshold values for optimal sensitivity of the neural network model (intercept: 0.75, Precision: 0.15, Recall: 1.000, F1-score: 0.26).

Depending on the NN output value, the group of participants with COPD was divided into subgroups of high (first NN output value ≥ 0.75) and low (first NN output value < 0.75) risk of developing AF (Table 4, Supplemental Table S5).

Patients at low risk of developing AF, compared with those at high risk, had less severe COPD (*p* < 0.0001), a shorter duration of smoking (*p* < 0.0001), and better FEV1 values (*p* < 0.01). In addition, participants at high risk for AF had larger heart chamber sizes (*p* < 0.01) as well as more severe mitral and tricuspid regurgitation (*p* < 0.0001). Changes in cardiac muscle geometry across the subgroups are presented in Supplemental Table S6.

Thus, changes in cardiac muscle geometry, described using the value obtained from the NN output, are associated with the development of AF in the group of patients with COPD.

#### 3.1.5. Selection of Threshold Values and Classification Metrics

Given the relatively small number of patients with AF, the predictive model was calibrated using a set of metrics. The calibration slope was 0.166, and the calibration intercept was −0.038. The Brier score was 0.0935, which is significantly lower than 0.25 (naive prediction based on outcome frequency) and confirms the model’s good discriminatory ability.

An analysis of threshold values ranging from 0.10 to 0.90 showed that the maximum value of the Youden index (0.686) is achieved at a threshold of 0.70. However, at this threshold, sensitivity is 1.000 (100%), while specificity is only 0.686, leading to an unacceptably high rate of false-positive classifications (at a threshold of 0.70, there would be 92 false-positive results versus 74 at a threshold of 0.75), which does not meet the objectives of clinical screening with limited resources. Given the clinical objective of minimizing missed cases of atrial fibrillation while maintaining an acceptable level of false positives, a threshold of >0.75 was selected, at which the sensitivity is 0.833 (15 out of 18 cases of AF detected), the specificity is 0.747, the positive predictive value (PPV) is 0.169, the negative predictive value (NPV) is 0.986, and the F1 score is 0.280. At this threshold, the confusion matrix is as follows: true positives—15, true negatives—219, false positives—74, false negatives—3.

Given the small number of true positive cases (*n* = 18), all confidence intervals were calculated using the bootstrap method with 2000 replicates. For a threshold > 0.75, the following results were obtained: sensitivity—0.833 (95% CI: 0.585–0.966), specificity—0.747 (95% CI: 0.694–0.796), positive predictive value—0.169 (95% CI: 0.105–0.248), negative predictive value—0.986 (95% CI: 0.969–0.997), positive likelihood ratio (LR+)—3.29 (95% CI: 2.48–4.21), negative likelihood ratio (LR–)—0.22 (95% CI: 0.07–0.57), F1 score—0.280 (95% CI: 0.179–0.396), accuracy—0.752 (95% CI: 0.701–0.800). The area under the ROC curve (AUC) was 0.8066 (95% CI: 0.6831–0.9301, *p* < 0.000001). The confidence interval for the threshold value, determined by the bootstrap method, is 0.71–0.78 (median threshold value in bootstrap samples—0.74), which confirms the stability of the selected threshold > 0.75.

### 3.2. Detection of Occult Arrhythmias Using a Neural Network

Given that the developed neural network reflects myocardial remodeling processes associated with arrhythmogenic atrial activity, it seems appropriate to use this model to detect occult atrial arrhythmias.

According to the consensus opinion, the diagnosis of AF during Holter monitoring requires an arrhythmia episode lasting at least 30 s [2]. A number of arrhythmias present a certain clinical challenge; these are similar to AF in morphology and associated with an increased risk of adverse events, but do not meet the formal diagnostic criteria for AF (so-called “micro-AF”). Such arrhythmias include group and multifocal supraventricular extrasystoles, short episodes of supraventricular tachycardia, and other rhythm disturbances that may be considered markers of undiagnosed AF [13].

#### 3.2.1. Participant Characteristics

Electronic medical records of 207 patients with COPD and no history of AF who underwent 24 h ECG monitoring were selected for the study.

The mean age of the study participants was 59.9 ± 17.9 years. According to the HATCH score, the participants were classified as being at moderate risk for atrial fibrillation (mean score: 3.93 ± 0.95; median: 4.0 [3.0–4.0]). The demographic and clinical characteristics of the study sample are presented in Table 5.

#### 3.2.2. Data from 24 h ECG Monitoring

All study participants underwent 24 h ECG monitoring with assessment of minimum, maximum, and average heart rates (HR) (Table 6). In addition, the total number of supraventricular and ventricular extrasystoles was analyzed, including their distribution by type (single, paired, and group) (Table 6).

Episodes of atrial fibrillation were detected in 4 (1.93%) study participants. Episodes of irregular supraventricular rhythm, defined as three or more consecutive complexes with a total duration of less than 30 s, were recorded in 50 (24.15%) participants.

#### 3.2.3. Comparison of Groups Based on NN Output

Based on the values obtained at the first output neuron of the NN, all study participants were divided into two groups. The first group (high risk of AF development, first NN output value > 0.75) consisted of 47 (22.7%) participants. The second (control) group (low risk of AF, first NN output value ≤ 0.75) consisted of 160 (77.3%) study participants.

A statistical comparison of the groups revealed no significant differences in a number of baseline parameters: smoking history, pack-years, as well as FEV1, the clinical indices ADO and CODEX, and the calculated risk of developing AF according to the HATCH scale.

However, the group with a high probability of developing AF was characterized by a more severe stage of COPD and more pronounced dyspnea according to the mMRC scale (*p* < 0.05) (Table 7).

**Figure 6:**
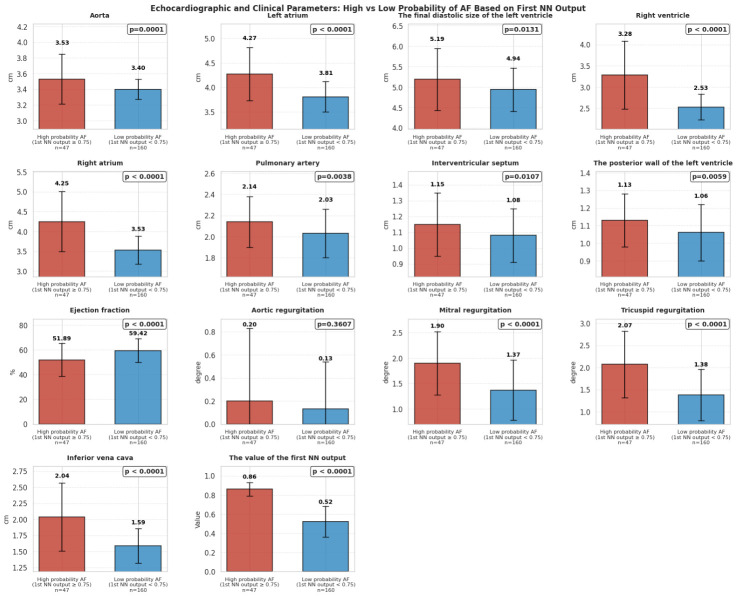
Main echocardiographic characteristics of study participants.

A comparative analysis of echocardiographic parameters revealed significant differences between the group with a high probability of developing AF and the low-risk group. The first group showed enlarged dimensions of all heart chambers, the ascending aorta, the pulmonary artery, and the inferior vena cava; increased left ventricular wall thickness; a lower ejection fraction; and more pronounced mitral and tricuspid regurgitation (all *p* < 0.05). The mean value of the first output of the neural network model was 0.86 ± 0.07 in the high-risk group versus 0.52 ± 0.16 in the control group (*p* < 0.0001) (Figure 6, Supplemental Table S7).

Analysis of 24 h ECG monitoring data revealed no statistically significant differences between groups in terms of heart rate (HR). The mean values of minimum, maximum, and average HR in the groups with a high probability of developing atrial fibrillation (AF) and in the control group were also comparable.

At the same time, participants in the group with a high probability of developing AF were characterized by a significantly higher frequency of supraventricular extrasystoles (SVEs). Statistically significant differences were observed for the total number of SVEs, as well as for single, paired, and group forms (*p* < 0.05). At the same time, no significant differences between the groups were found in the total number of ventricular premature beats (VPBs) or their distribution by grade (Supplemental Table S8).

The groups were comparable in terms of age, sex, prevalence of cardiovascular diseases (MI, stroke, hypertension), and baseline COPD treatment. In the high-risk group for AF, diabetes mellitus (*p* < 0.05) and stage IV COPD (*p* = 0.02) were significantly more common. According to Holter monitoring data, all cases of AF (8.51% vs. 0%; *p* < 0.0001) and, more frequently, group supraventricular extrasystoles (46.81% vs. 17.5%; *p* < 0.0001) were detected in the high-risk group. The combined arrhythmic endpoint (AF + GSE) was recorded in 55.32% versus 17.5% of participants, respectively (*p* < 0.0001) (Supplemental Table S9).

#### 3.2.4. ROC Analysis

**Figure 7:**
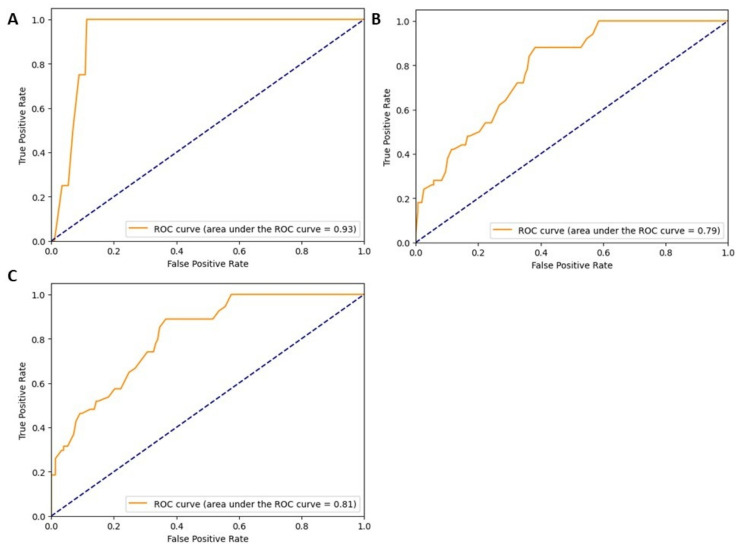
Results of the ROC analysis of the detection rate during 24 h ECG monitoring as a function of the value of the first output of the neural network. (**A**) atrial fibrillation; (**B**) group supraventricular extrasystoles; (**C**) combined endpoint (atrial fibrillation or group supraventricular extrasystoles). The dashed diagonal line represents the reference line where the area under the curve (AUC) is 0.5, indicating no discriminative ability.

To evaluate the diagnostic accuracy of the developed neural network model in detecting various types of atrial arrhythmias, a ROC analysis was performed (a nonparametric evaluation method using MedCalc software, version 22.016). Figure 7 shows the ROC curves for three clinical endpoints. The area under the ROC curve was:

1. For the detection of atrial fibrillation during 24 h ECG monitoring based on the value of the first output of the neural network: 0.93.
2. For detecting group supraventricular extrasystoles during 24 h ECG monitoring based on the value of the first output of the neural network: 0.79.
3. For detecting a combined event (atrial fibrillation or group supraventricular extrasystoles) during 24 h ECG monitoring based on the value of the first output of the neural network: 0.81.

Based on the results obtained, patients with COPD, sinus rhythm, and a first-output value of the neural network > 0.75 may be considered candidates for further rhythm monitoring to assess for possible AF.

An analysis of precision–recall metrics for various arrhythmic endpoints is presented in Supplemental Table S10. The optimal threshold value of the first output neuron for detecting clustered supraventricular extrasystoles (“micro-AF”) was 0.61 (sensitivity 88%, precision 42%, F1-score 0.57). For detecting concealed atrial fibrillation, a threshold of 0.85 provides 100% sensitivity (all cases are detected), but the precision is only 15% (positive predictive value 0.15), which indicates a high rate of false positives.

Thus, the presented model can be used in AF screening in combination with other methods to identify indications for long-term ECG monitoring to detect occult arrhythmias. By adjusting the threshold values, the model’s sensitivity and specificity can be tailored to specific tasks, which represents a promising direction for future research.

## 4. Discussion

This study focuses on the development and validation of a neural network diagnostic model based on transthoracic echocardiography to address two clinical challenges in patients with COPD: prediction of the risk of atrial fibrillation (AF); detection of occult (silent) supraventricular arrhythmias (including “micro-AF”) based on 24 h ECG monitoring data. The main result of the current study is the development and validation of a neural network model based on 13 standard echocardiographic parameters, allowing for the quantitative assessment of the degree of structural and functional myocardial remodeling in patients with COPD. At a threshold value of the first output neuron > 0.75, the model stratifies patients with COPD into high- and low-risk groups for the development of AF: the AF incidence in the high-risk group was 14.8% versus 0% in the low-risk group (*p* = 0.0073). In addition, the model demonstrated high diagnostic accuracy in detecting occult supraventricular arrhythmias in patients with COPD without a known history of AF.

A significant body of data has been accumulated in the international literature regarding the association between COPD and atrial fibrillation. According to data from the Swedish registry for 1995–2008, AF significantly increases all-cause mortality, and the presence of COPD as a comorbid condition in such patients contributes substantially to an unfavorable prognosis [14]. Many risk factors for cardiovascular disease, such as hypertension, dyslipidemia, chronic kidney disease, obesity, and a number of others, are quite common in the COPD patient population [15]. Based on a cross-sectional analysis of 1,204,100 patients over the age of 35, Feary et al. demonstrated that COPD significantly increases the risk of developing cardiovascular disease (OR 4.98; 95% CI 4.85–5.81; *p* < 0.001) and stroke (OR 3.34; 95% CI 3.21–3.48; *p* < 0.001) [16].

In the current study, AF developed in 18 (5.79%) participants during the 24-month follow-up period; the median time to AF onset was 6.5 [5.25–10.5] months. At the same time, a trend toward more frequent AF development was observed in the group of participants with COPD, although the differences did not reach statistical significance. Similar data were obtained in the CHARGE-AF study: the mean follow-up period was 3.5 ± 1.7 years, and the mean age of participants was 65.5 ± 11.24 years. AF developed in 4.7% of participants. The prevalence of COPD was significantly higher among patients who developed AF compared to those who remained in sinus rhythm [17].

Electrophysiological and structural myocardial remodeling represent a key link between COPD and AF. Echocardiography is a widely available method that allows for reliable assessment of cardiac structure and function. Several studies confirm the association between myocardial remodeling in patients with COPD and the development of AF. For example, in one study, left ventricular diastolic dysfunction was detected in 88% of participants with COPD (*p* < 0.05); these same patients also exhibited more pronounced signs of right ventricular overload (*p* < 0.05) [18]. In a study by Roh et al., changes in pulmonary vein morphology were frequently detected in patients with chronic bronchopulmonary disease, and these were associated with arrhythmogenicity in 40% of patients; furthermore, extrapulmonary foci of arrhythmogenic activity were more common in these patients [19]. Thus, according to Hirose T. et al., when evaluating left atrial function, the group of patients with established AF differed significantly in left atrial active ejection fraction (16 ± 5% vs. 28 ± 8%, *p* < 0.001), myocardial strain rate (−0.9 ± 0.2 vs.−1.4 ± 0.5, *p* < 0.001), and left atrial volume index (59 ± 12 vs. 46 ± 16 mL/m^2^, *p* < 0.001) [20].

In the study by De Vos et al. (*n* = 249, follow-up period 1.86 ± 0.79 years), AF developed in 6% of patients. The PA-TDI interval was significantly longer in the group of patients who developed AF (172 ± 25 vs. 150 ± 20 ms; *p* = 0.001) and was an independent predictor (OR 1.375; *p* = 0.027). The two-year incidence of AF was 33% with PA-TDI > 190 ms and 0% with PA-TDI < 130 ms (*p* = 0.002) [21]. In addition to the echocardiographic parameters used in our model, clinical risk factors such as obesity also contribute to atrial remodeling and the risk of developing AF. According to a review by Na et al., obesity contributes to the development of AF by increasing left atrial size, causing epicardial fat accumulation, and leading to diastolic dysfunction, which predisposes to heart failure [22].

There are a number of validated clinical scales that assess the risk of developing AF. The most well-known are the Framingham scale, the ARIC study scale, as well as the CHARGE-AF, C2HEST, and HATCH scales [23,24,25,26,27]. These clinical scales have demonstrated fairly high prognostic accuracy (AUC 0.72–0.78). It is worth noting that only two of them (CHARGE-AF, C2HEST) consider COPD as an independent risk factor for the development of AF.

There are also a number of AF prediction models based on algorithms using artificial intelligence [28]. Although these models have better predictive performance compared to validated clinical scales (AUC 0.80–0.83), their use in some cases requires the input of fairly large volumes of standardized data, and sometimes the correction of this data during patient follow-up [29]. Russian researchers developed a neural network model for predicting AF in patients with coronary artery disease (CAD), incorporating echocardiographic and anthropometric parameters, social factors, and an assessment of coronary artery status. The model demonstrated high sensitivity and specificity in men with CAD [30]. Our model demonstrates comparable prognostic performance when using only standard echocardiographic parameters, making it more accessible for widespread use. Additionally, the model demonstrated high accuracy in detecting silent arrhythmias (AUC 0.93), which expands its clinical potential.

Our neural network model proved capable of identifying patients at high risk not only for overt AF but also for silent supraventricular arrhythmias, including runs of supraventricular premature beats and brief episodes of supraventricular tachycardia (“micro-AF”). In the STROKESTOP II study (3763 participants without previously diagnosed AF), episodes of “micro-AF” were detected in 221 (6%) individuals. During long-term continuous ECG monitoring in the “micro-AF” subgroup (*n* = 196), atrial fibrillation was verified in 26 (13%) participants, which significantly exceeded the rate in the control group (*n* = 250), whereas AF was detected in only 7 (3%) individuals (*p* < 0.001) [13].

### Study Limitations

It is important to note that the current study has certain limitations. The study was conducted at a single center (Lipetsk City Hospital), and all participants were permanent residents of the Lipetsk region of Caucasian descent. This may limit the generalizability of the results to other ethnic groups, geographic regions, and medical institutions with different patient demographics and clinical practices. Another limitation is that the first phase of the study (neural network development and training) was conducted retrospectively based on an analysis of medical records. Third, during the validation phase (phase 2), atrial fibrillation developed in only a small number of participants. The limited number of events affects the precision of our estimates (wide confidence intervals) and prevents us from conducting more complex subgroup analyses. Furthermore, this increases the risk of model overfitting, despite our efforts to split the samples into training, validation, and test sets. Despite the use of predefined inclusion and exclusion criteria, selection bias cannot be completely ruled out, especially in stage 3, which included only patients with documented COPD referred for 24 h ECG monitoring. To detect occult atrial arrhythmias (stage 3), we used a single 24 h Holter monitoring session. This method may miss very brief episodes of AF or those occurring outside the monitoring period. The neural network model was developed, internally validated, and tested on single-center cohorts. However, the low slope of the calibration curve and the low positive predictive value necessitate further validation of the model on a larger, independent cohort for clinical implementation. Despite the use of separate training, validation, and test sets, the relatively complex architecture combined with a moderate sample size for development creates a risk of overfitting. In addition, baseline treatment for COPD (bronchodilators, inhaled corticosteroids, oxygen therapy) and cardiovascular comorbidities (antihypertensive drugs, statins, antiplatelet agents) was recorded but not standardized.

## 5. Conclusions

Thus, in the course of this study, the developed neural network model demonstrated high predictive value in assessing the risk of atrial fibrillation. The model allowed for the quantitative characterization of myocardial remodeling processes via the value of the first output neuron, with a threshold value of 0.75 significantly dividing patients into high- and low-risk groups for AF. In patients with COPD, more pronounced structural and functional changes in the heart were identified, along with a twofold higher incidence of AF. Logistic regression models based on the neural network’s output proved statistically significant for both groups, unlike models using individual echocardiographic parameters, confirming the advantage of an integrated approach.

The developed neural network model (MLP with 4 hidden layers of 130 neurons each), integrating 13 echocardiographic parameters, provides a quantitative assessment of myocardial remodeling with high accuracy in classifying AF: Accuracy = 0.81, AUC = 0.80 (95% CI 0.71–0.87). The logistic regression model of the relationship between AF development and the NN output value is statistically significant (*p* < 0.0001).

In patients with COPD without a history of AF in the group with an NN output > 0.75, the following were more frequently recorded: AF episodes: 8.51% versus 0% (*p* < 0.0001); group supraventricular extrasystoles: 46.81% vs. 17.5% (*p* < 0.0001); combined endpoint (AF + SVE): 55.32% vs. 17.5% (*p* < 0.0001). The area under the ROC curve for detecting asymptomatic AF based on 24 h ECG monitoring data, depending on the NN output value, was 0.93.

Thus, the presented NN model may help identify COPD patients who could benefit from intensified rhythm monitoring for the early detection of AF. However, external validation in independent multicenter cohorts is required prior to clinical implementation.

## Figures and Tables

**Table 1:** Baseline characteristics of patients in the validation cohort.

Characteristic	Value
Age and gender distribution of the sample, risks of developing AF	
Mean age of participants, years	64.27 ± 14.27
Women	148 (47.59%)
Median follow-up, months	18.0 [14.0; 21.0]
Participants who developed AF	18 (5.79%)
Median time to AF onset, months	6.5 [5.25–10.5]
Participants who died during the study	28 (9.0%)
Risk of developing AF according to the HATCH scale	1.85 ± 1.14
Comorbidities	
Malignant neoplasms	14 (4.5%)
Hypothyroidism	9 (2.89%)
Diabetes mellitus	39 (12.54%)
History of stroke	10 (3.22%)
Cerebrovascular disease	46 (14.79%)
Dilated cardiomyopathy	7 (2.25%)
COPD	99 (31.83%)
Aortic valve stenosis	3 (0.96%)
Congestive heart failure	18 (5.79%)
Gastric ulcer	1 (0.32%)
Moderate or severe chronic kidney disease (creatinine > 270 μmol/L)	1 (0.32%)
Charlson Comorbidity Index	2.63 ± 1.54
Causes of death	
Chronic heart failure	13 (46.43%)
Malignant neoplasms	5 (17.86%)
Chronic respiratory failure	3 (10.71%)
Ischemic (cardioembolic) stroke	3 (10.71%)
Chronic cerebral insufficiency	2 (7.14%)
Gastrointestinal bleeding	1 (3.57%)
Hemorrhagic stroke	1 (3.57%)
Total due to cardiovascular disease	19 (67.86%)

**Table 2:** Characteristics of study participants stratified by COPD status.

Characteristic	Patients With COPD (*n* = 99)	Patients Without COPD (*n* = 212)	Significance of Differences
Mean age of participants, years	69.55 ± 5.73	61.81 ± 16.79	*p* = 0.0002
Women	16 (16.16%)	132 (62.26%)	*p* < 0.0001
Median follow-up, months	19 [17–21]	16 [14–21]	*p* < 0.0001
Participants who developed AF	8 (8.08%)	10 (4.72%)	*p* = 0.2967
Median time to AF onset, months	7.5 [5.75–11.25]	6 [5.25–8.75]	*p* = 0.5764
Participants who died during the study	7 (7.07%)	21 (9.91%)	*p* = 0.014
Median time to death, months	16.5 [15.0–20.5]	5.0 [3.0–10.0]	*p* < 0.0001
Arterial hypertension	94 (94.94%)	196 (92.45%)	*p* = 0.4770
Cancer	4 (4.04%)	10 (4.72%)	*p* = 1.0
Hypothyroidism	0	9 (4.25%)	*p* = 0.0619
Diabetes mellitus	7 (7.07%)	32 (15.09%)	*p* = 0.0646
Cerebrovascular disease	10 (10.1%)	36 (16.98%)	*p* = 0.125
Ischemic stroke	3 (3.03%)	7 (3.30%)	*p* = 1.0
Dilated cardiomyopathy	0	7 (3.30%)	*p* = 0.1017
Aortic valve stenosis	0	3 (1.42%)	*p* = 0.5540
Congestive heart failure	3 (3.03%)	15 (7.08%)	*p* = 0.1972
Stomach ulcer	0	1 (0.47%)	*p* = 1.0
Moderate or severe chronic kidney disease (creatinine > 270 mmol/L)	0	1 (0.47%)	*p* = 1.0
Charlson Comorbidity Index	3.45 ± 1.05	2.31 ± 1.58	*p* < 0.0001
Death from cardiovascular diseases	4 (4.04%)	14 (6.60%)	*p* = 0.4439
Death from heart failure	3 (3.03%)	9 (4.25%)	*p* = 0.7583

**Table 3:** Results of logistic regression analysis for AF development based on NN output value.

Group	Coefficient	Statistical Significance
Patients with COPD (*n* = 99)	−3.06 ± 0.59	*p* < 0.0001
Patients without COPD (*n* = 212)	−4.11 ± 0.48	*p* < 0.0001
The entire sample	−3.72 ± 0.36	*p* < 0.0001

**Table 4:** The main characteristics of high and low risk groups of AF according to the NN data.

Characteristic	Group Low Risk of AF with COPD, First NN Output Value < 0.75 (*n* = 45)	Group High Risk of AF with COPD, First NN Output Value ≥ 0.75 (*n* = 54)	Statistical Significance
The average age of the participants, years	67.6 ± 6.39	71.17 ± 4.58	*p* = 0.0017
Women	11 (24.44%)	5 (9.26%)	*p* = 0.0554
Median follow-up, months	19.0 [17.0; 21.0]	19.0 [16.0; 22.0]	*p* = 0.8546
The risk of developing AF on the HATCH scale	3.18 ± 0.44	3.26 ± 0.59	*p* = 0.4456
Participants who developed AF	0	8 (14.81%)	*p* = 0.0073
Number of deaths during the study	2 (4.44%)	5 (9.26%)	*p* = 0.4503
Median onset of death, months	20.5 [19.75; 21.25]	17.0 [16.0; 18.0]	*p* < 0.001

**Table 5:** Baseline characteristics of patients in the arrhythmia detection cohort.

Parameter	Mean Median	Normality of Distribution
Demographic characteristics		
Age, years	59.91 ± 17.91 64.0 [50.0; 72.5]	*p* < 0.0001
Women	25 (12.1%)	
COPD course		
HATCH score	3.93 ± 0.95 4.0 [3.0; 4.0]	*p* < 0.0001
Duration of smoking, years	33.67 ± 8.33 32.0 [30.0; 41.0]	*p =* 0.0002
Pack-years	34.19 ± 8.79 34.0 [29.0; 42.0]	*p =* 0.0017
FEV1, %	54.24 ± 11.47 58.0 [55.0; 60.0]	*p* < 0.0001
Dyspnea severity according to mMRC	1.3 ± 0.71 1.0 [1.0; 2.0]	*p* < 0.0001
Number of COPD exacerbations per year	0.63 ± 0.73 0.0 [0.0; 1.0]	*p* < 0.0001
ADO scale scores	4.29 ± 1.39 4.0 [3.0; 5.0]	*p* < 0.0001
Scores on the CODEX scale	2.29 ± 1.77 2.0 [1.0; 3.0]	*p* < 0.0001
Transthoracic echocardiography, AF prognosis		
Aorta, cm	3.54 ± 0.14 3.5 [3.4; 3.6]	*p =* 0.0001
Left atrium, cm	3.91 ± 0.42 3.8 [3.6; 4.1]	*p* < 0.0001
LVEDD, cm	5.0 ± 0.6 4.9 [4.6; 5.1]	*p =* 0.0131
Right ventricle, cm	2.7 ± 0.56 2.5 [2.4; 2.7]	*p* < 0.0001
Right atrium, cm	3.7 ± 0.56 3.4 [3.4; 3.6]	*p* < 0.0001
Pulmonary artery, cm	2.14 ± 0.24 2.0 [1.9; 2.2]	*p =* 0.0038
IVS, cm	1.09 ± 0.18 1.0 [1.0; 1.2]	*p =* 0.0107
LVPW, cm	1.07 ± 0.16 1.0 [1.0; 1.2]	*p =* 0.0059
LVEF, %	57.71 ± 10.91 61.0 [57.0; 66.0]	*p* < 0.0001
AR, degree	0.15 ± 0.47 0.0 [0.0; 0.0]	*p =* 0.3607
MR, degree	1.49 ± 0.64 1.0 [1.0; 2.0]	*p* < 0.0001
TR, degree	1.54 ± 0.69 1.0 [1.0; 2.0]	*p* < 0.0001
Inferior vena cava, cm	1.3 ± 0.71 1.0 [1.0; 2.0]	*p* < 0.0001
Value of the first output of the neural network	0.6 ± 0.21 0.61 [0.47; 0.72]	*p* < 0.0001

Note. FEV1—forced expiratory volume in 1 s; COPD—chronic obstructive pulmonary disease; AR—aortic regurgitation; LVPW—left ventricular posterior wall; LVEDD—left ventricular end-diastolic diameter; LV—left ventricle; IVS—interventricular septum; MR—mitral regurgitation; NN—neural network; TR—tricuspid regurgitation; TTE—transthoracic echocardiography; EF—ejection fraction; AF—atrial fibrillation.

**Table 6:** Key indicators of 24 h ECG monitoring of study participants.

Parameter	Mean ± SD; Median [IQR]	*p*-Value for Normality
Minimum HR per minute	56.65 ± 32.1 55.0 [47.5; 60.0]	*p* < 0.0001
Maximum heart rate per minute	108.12 ± 23.34 106.0 [90.5; 120.0]	*p* = 0.0031
Average heart rate per minute	71.66 ± 13.94 69.0 [62.0; 82.0]	*p* = 0.0004
Supraventricular extrasystoles (total)	440.49 ± 1337 18.0 [5.0; 161.5]	*p* < 0.0001
Isolated supraventricular extrasystoles	416.1 ± 1233 17.0 [5.0; 145.5]	*p* < 0.0001
Paired supraventricular extrasystoles	20.08 ± 136.83 0.0 [0.0; 3.0]	*p* < 0.0001
Group supraventricular extrasystoles	3.18 ± 15.75 0.0 [0.0; 1.0]	*p* < 0.0001
Ventricular premature beats (total)	428.34 ± 2811 4.0 [0.0; 32.5]	*p* < 0.0001
Isolated ventricular premature beats	420.91 ± 2803 3.0 [0.0; 30.0]	*p* < 0.0001
Paired ventricular premature beats	3.94 ± 29.52 0.0 [0.0; 0.0]	*p* < 0.0001

**Table 7:** Comparison of clinical and echocardiographic characteristics between high- and low-risk groups based on NN output.

Parameter	Participants with a High Probability of AF, Value of the First Output of the NN ≥ 0.75 (*n* = 47)	Participants with a Low Probability of AF, Value of the First Output of the NN < 0.75 (*n* = 160)	Significance of Differences
Age, years	63.79 ± 13.65	58.78 ± 18.86	*p* = 0.0916
Women	9 (19.12%)	16 (10.0%)	*p* = 0.0427
HATCH score	4.06 ± 1.01	3.89 ± 0.93	*p* = 0.2643
COPD stage	3.3 ± 0.69	3.05 ± 0.64	*p* = 0.0232
Duration of smoking, years	34.72 ± 9.33	33.36 ± 8.01	*p* = 0.3257
Pack-years index	34.77 ± 9.59	34.02 ± 8.56	*p* = 0.6094
FEV1	54.21 ± 10.75	54.25 ± 11.71	*p* = 0.9845
Severity of dyspnea according to mMRC	1.53 ± 0.72	1.24 ± 0.70	*p* = 0.0121
Number of COPD exacerbations per year	0.81 ± 0.77	0.58 ± 0.71	*p* = 0.0608
ADO scale scores	4.45 ± 1.32	4.24 ± 1.41	*p* = 0.3655
CODEX scale score	2.28±1.54	2.30 ± 1.84	*p* = 0.9369

## Data Availability

The raw data supporting the conclusions of this article will be made available by the authors on request.

## Supplementary Materials

The following supporting information can be downloaded at: https://www.mdpi.com/article/10.3390/diseases14060206/s1, Table S1: Training parameters, metrics, and risk criteria for the neural network; Table S2: Clinical and functional characteristics of patients with COPD; Table S3: Echocardiographic parameters of the study participants; Table S4: Echocardiographic parameters of study participants according to COPD stage; Table S5: Additional characteristics of participants with COPD; Table S6: Echocardiographic parameters of high- and low-risk groups for AF according to NN data; Table S7: Main echocardiographic characteristics of study participants; Table S8: Key indicators of daily ECG monitoring of study participants; Table S9: Selected characteristics of study participants; Table S10: Performance metrics of the neural network for detecting different arrhythmic endpoints.
