# Supplementary material for: Development and Validation of a Neural Network Model for Predicting Atrial Fibrillation and Detecting Silent Arrhythmias in Patients with Chronic Obstructive Pulmonary Disease Based on Echocardiography Data
Source: Diseases. 2026 Jun 9;14(6):206. doi: 10.3390/diseases14060206 (PMC13298696; doi:10.3390/diseases14060206)
Supplement: Supplementary file 1 [file diseases-14-00206-s001.zip › diseases-4333162-supplementary.pdf]

Supplemental Table S1. Training parameters, metrics, and risk criteria for the neural network

Characteristic	Value
Total sample	684 patients (292 without AF, 392 with AF)
Training	500 vectors (200 without AF, 300 with AF) x_train, y_train
Validation	84 vectors (28 without AF, 56 with AF) x_val, y_val
Test	100 vectors (34 without AF, 66 with AF) x_test, y_test
Normalization	$r = (n - min)/(max - min)$, range [0, 1]
Data format	Two-dimensional matrix: 684 vectors × 13 values
Accuracy	0.81
Precision	0.87
Recall	0.83
AUC ROC	0.80 (95% CI 0.71–0.87)
High risk	Value of the first NN output > 0.75
Low risk	Value of the first output of the neural network ≤ 0.75

Supplemental Table S2. Clinical and functional characteristics of patients with COPD

Characteristic	Value
COPD stage, median	3.0 [3.0–4.0]
Smoking history, years	33.43 ± 8.85
Pack-years	33.48 ± 8.96
FEV1, %	54.71±11.21
Tiffeneau index, %	59.23 ± 8.87
Shortness of breath (mMRC)	1.21±0.67
Inhaled glucocorticosteroids	50 (50.51%)
Beta-adrenergic agonists	75 (75.76%)
M-cholinolytics	66 (66.67%)
ADO scale scores	3.94 ± 1.38
CODEX scale scores	1.74 ± 1.57

Supplemental Table S3. Echocardiographic parameters of the study participants

Characteristic	Patients with COPD (n=99)	Patients without COPD (n=212)	Statistical significance
Aorta, cm	3.52±0.33	3.24±0.35	p<0.0001
Left atrium, cm	4.05±0.45	3.77 ± 0.48	p<0.0001
Final diastolic size of the left ventricle, cm	5.15±0.47	4.82 ± 0.64	p<0.0001
Right ventricle, cm	3.56±0.71	2.66 ± 0.38	p<0.0001
Right atrium, cm	4.12 ± 0.72	3.56 ± 0.40	p<0.0001
Pulmonary artery, cm	2.32 ± 0.36	2.18 ± 0.24	p=0.0043
Interventricular septum, cm	1.19±0.19	1.16 ± 0.21	p=0.0914
The posterior wall of the left ventricle, cm	1.17 ± 0.22	1.09 ± 0.16	p=0.0027
Ejection fraction, %	59.92±4.62	61.49 ± 8.79	p=0.0955
Aortic regurgitation, degree	0.04±0.25	0.11 ± 0.39	p=0.0594
Mitral regurgitation, degree	1.69±0.51	1.51 ± 0.50	p=0.0004
Tricuspid regurgitation, degree	1.67±0.64	1.34 ± 0.57	p<0.0001
Inferior vena cava, cm	1.85±0.37	1.69 ± 0.36	p=0.0001

Supplemental Table S4. Echocardiographic parameters of study participants according to COPD stage

Characteristic	COPD Stage II (n=18)	COPD III (n=55)	COPD IV (n=26)	Statistical significance
Aorta, cm	3.36±0.37	3.53±0.33	3.61±0.26	$p^{1,2}$ =0.0742<br>$p^{1,3}$ =0.0123<br>$p^{2,3}$ =0.261
Left atrium, cm	3.74 ± 0.39	4.03 ± 0.41	4.3 ± 0.44	$p^{1,2}$= 0.0083<br>$p^{1,3}$ =0.0001<br>$p^{2,3}$ =0.0087
The final diastolic size of the left ventricle, cm	5.03±0.43	5.12 ± 0.47	5.31±0.47	$p^{1,2}$= 0.3964<br>$p^{1,3}$ =0.051<br>$p^{2,3}$ =0.1033
Right ventricle, cm	2.93±0.62	3.61 ± 0.68	3.87±0.56	$p^{1,2}$= 0.0009<br>$p^{1,3}$ <0.0001<br>$p^{2,3}$ =0.3745
Right atrium, cm	3.65±0.55	4.17 ± 0.73	4.33±0.69	$p^{1,2}$ =0.0119<br>$p^{1,3}$ =0.0013<br>$p^{2,3}$ =0.3745
Pulmonary artery, cm	2.19 ± 0.42	2.33 ± 0.33	2.38 ± 0.32	$p^{1,2}$= 0.0872<br>$p^{1,3}$ = 0.0928<br>$p^{2,3}$ = 0.4751
Interventricular septum, cm	1.12±0.13	1.21 ± 0.21	1.19±0.18	$p^{1,2}$= 0.0823<br>$p^{1,3}$ =0.1355<br>$p^{2,3}$ =0.6661
The posterior wall of the left ventricle, cm	1.09±0.14	1.16 ± 0.2	1.23 ± 0.29	$p^{1,2}$= 0.3185<br>$p^{1,3}$ =0.0631<br>$p^{2,3}$ =0.19
Ejection fraction, %	61.22±5.13	59.2 ± 4.39	60.54±4.62	$p^{1,2}$= 0.0874<br>$p^{1,3}$ =0.6466<br>$p^{2,3}$ =0.2116
Aortic regurgitation, degree	0.19 ± 0.57	0.01±0.07	0.0±0.0	$p^{1,2}$= 0.0836<br>$p^{1,3}$ =0.0889<br>$p^{2,3}$ =0.4952
Mitral regurgitation, degree	1.19±0.39	1.74 ± 0.51	1.92 ± 0.34	$p^{1,2}$= 0.0001<br>$p^{1,3}$ <0.0001<br>$p^{2,3}$ =0.0926
Tricuspid regurgitation, degree	1.28 ± 0.57	1.7 ± 0.66	1.88 ± 0.5	$p^{1,2}$= 0.0128<br>$p^{1,3}$ =0.0006<br>$p^{2,3}$ =0.2115
Inferior vena cava, cm	1.49±0.32	1.89 ± 0.36	2.02 ± 0.25	$p^{1,2}$= 0.0001<br>$p^{1,3}$ <0.0001<br>$p^{2,3}$= 0.0946

Supplemental Table S5. Additional characteristics of participants with COPD

**Characteristic**	**Group low risk of AF with COPD, first NN output value < 0.75 (n=45)**	**Group high risk of AF with COPD, first NN output value ≥ 0.75 (n=54)**	**Statistical significance**
COPD stage, median	3.0 [2.0; 3.0]	3.0 [3.0; 4.0]	p<0.0001
Duration of smoking	30.0 [23.0; 33.0]	37.0 [30.0; 43.0]	p<0.0001
Pack-years index	29.0 [22.0; 33.0]	37.0 [32.0; 44.0]	p<0.0001
FEV1, %	60.0 [56.0; 64.0]	56.0 [45.25; 59.0]	p=0.0012
The Tiffno Index, %	59.0 [54.0; 64.0]	62.0 [56.0; 67.0]	p=0.5006
mMRC-scale	1.0 [1.0; 2.0]	1.0 [1.0; 1.0]	p=0.2908
Beta-adrenergic agonists	33 (73.33%)	42 (77.78%)	p=0.6437
Inhaled glucocorticosteroids	27 (60.0%)	23 (42.59%)	p=0.1074
M-cholinolytics	26 (57.78%)	40 (74.07%)	p=0.0932
Points on the ADO scale	3.0 [3.0; 5.0]	4.0 [3.0; 4.0]	p=0.1775
Points on the CODEX scale	1.0 [1.0; 3.0]	1.0 [1.0; 2.0]	p=0.1512

Supplemental Table S6. Echocardiographic parameters of high- and low-risk groups for AF according to NN data

Characteristic	Group<br>Low risk of AF with COPD, first NN output value < 0.75 (n=45)	Group<br>High risk of AF with COPD, first NN output value ≥ 0.75 (n=54)	Statistical significance
Aorta, cm	3.4±0.37	3.62±0.26	p=0.0005
Left atrium, cm	3.74±0.36	4.3 ± 0.35	p<0.0001
The final diastolic size of the left ventricle, cm	4.99 ± 0.44	5.29 ± 0.45	p=0.0015
Right ventricle, cm	2.99±0.64	4.03 ± 0.29	p<0.0001
Right atrium, cm	3.7±0.5	4.47 ± 0.7	p<0.0001
Pulmonary artery, cm	2.18 ± 0.32	2.43 ± 0.33	p=0.0003
Interventricular septum, cm	1.12±0.15	1.25 ± 0.2	p=0.0004
The posterior wall of the left ventricle, cm	1.1±0.15	1.23 ± 0.25	p=0.0023
Ejection fraction, %	60.36±4.98	59.56 ± 4.31	p=0.3937
Aortic regurgitation, degree	0.09±0.37	0.0±0.0	p=0.0833
Mitral regurgitation, degree	1.38 ± 0.56	1.94 ± 0.27	p<0.0001
Tricuspid regurgitation, degree	1.38 ± 0.58	1.92 ± 0.58	p<0.0001
Inferior vena cava, cm	1.59±0.33	2.07 ± 0.24	p<0.0001

Supplemental Table S7. Main echocardiographic characteristics of study participants

Characteristic	Participants with a high probability of AF, first NN output value ≥ 0.75 (n=47)	Participants with a low probability of AF, first NN output value < 0.75 (n=160)	Statistical significance
Aorta, cm	3.53±0.32	3.4 ± 0.13	p=0.0001
Left atrium, cm	4.27 ± 0.54	3.81 ± 0.31	p<0.0001
The final diastolic size of the left ventricle, cm	5.19 ± 0.76	4.94 ± 0.53	p=0.0131
Right ventricle, cm	3.28±0.8	2.53 ± 0.3	p<0.0001
Right atrium, cm	4.25 ± 0.76	3.53 ± 0.35	p<0.0001
Pulmonary artery, cm	2.14 ± 0.24	2.03 ± 0.23	p=0.0038
Interventricular septum, cm	1.15±0.2	1.08 ± 0.17	p=0.0107
The posterior wall of the left ventricle, cm	1.13 ± 0.15	1.06 ± 0.16	p=0.0059
Ejection fraction, %	51.89±13.37	59.42 ± 9.46	p<0.0001
Aortic regurgitation, degree	0.2 ± 0.63	0.13 ± 0.41	p=0.3607
Mitral regurgitation, degree	1.9 ± 0.62	1.37 ± 0.59	p<0.0001
Tricuspid regurgitation, degree	2.07 ± 0.75	1.38 ± 0.58	p<0.0001
Inferior vena cava, cm	2.04 ± 0.53	1.59 ± 0.27	p<0.0001
The value of the first NN output	0.86 ± 0.07	0.52 ± 0.16	p<0.0001

Supplemental Table S8. Key indicators of daily ECG monitoring of study participants

Characteristic	Participants with a high probability of AF, first NN output value ≥ 0.75 (n=47)	Participants with a low probability of AF, first NN output value < 0.75 (n=160)	Statistical significance
Minimum heart rate per minute	55.77 ± 13.64	56.91 ± 35.79	p=0.8843
Maximum heart rate per minute	104.62 ± 19.22	109.14 ± 24.38	p=0.4461
Mean heart rate per minute	71.04±15.27	71.84 ± 13.57	p=0.6807
Supraventricular extrasystoles (total)	592.26 ± 994.72	395.91 ± 1421.42	p=0.001
Single supraventricular extrasystoles	568.55 ± 955.96	371.31 ± 1302.5	p=0.0019
Paired supraventricular extrasystoles	19.06 ± 42.68	20.38 ± 154.04	p=0.0016
Grouped supraventricular extrasystoles (salvos/runs)	5.51 ± 14.08	2.49 ± 16.19	p<0.0001
Ventricular extrasystoles (total)	73.34 ± 147.71	532.62 ± 3191.94	p=0.2345
Single ventricular extrasystoles	59.28 ± 127.41	527.14 ± 3181.61	p=0.4201
Paired ventricular extrasystoles	0.94 ± 3.14	4.82 ± 33.51	p=0.4919

Supplemental Table S9. Selected characteristics of study participants

Parameter	Participants with a high probability of AF, first output NN value ≥ 0.75 (n=47)	Participants with low probability of AF, value of the first NN output < 0.75 (n=160)	Significance of differences
Male	38 (80.85%)	144 (90.0%)	p=0.0427
History of myocardial infarction	9 (19.15%)	18 (11.25%)	p=0.2431
History of stroke	3 (6.38%)	9 (5.62%)	p=1.0
Hypertension	41 (87.23%)	123 (76.88%)	p=0.182
Malignant neoplasms	1 (2.13%)	3 (1.88%)	p=1.0
Diabetes	13 (27.66%)	22 (13.75%)	p=0.0439
Participants with stage II COPD	6 (12.77%)	28 (17.5%)	p=0.5849
Participants with stage III COPD	21 (44.68%)	94 (58.75%)	p=0.1237
Participants with stage IV COPD	20 (42.55%)	38 (23.75%)	p=0.0193
Fewer than 1 COPD exacerbation during the year	19 (40.43%)	88 (55.0%)	p=0.1114
1 COPD exacerbation during the year	18 (38.3%)	51 (31.87%)	p=0.5188
2 or more COPD exacerbations during the year	10 (21.28%)	21 (13.12%)	p=0.2525
ICS therapy	31 (65.96%)	78 (48.75%)	p=0.056
Beta-adrenergic agonist therapy	37 (78.72%)	121 (75.62%)	p=0.8071
M-cholinolytic therapy	37 (78.72%)	103 (64.38%)	p=0.0947
Episodes of AF	4 (8.51%)	0	p<0.0001
Episodes of group supraventricular extrasystoles (GSE)	22 (46.81%)	28 (17.5%)	p<0.0001
Combined endpoint (AF episodes + GSE)	26 (55.32%)	28 (17.5%)	p<0.0001

Supplemental Table S10. Performance metrics of the neural network for detecting different arrhythmic endpoints

**Parameter**	**Precision**	**Recall**	**F1 score**	**Threshold**	**Area under the PR curve (AUPRC)**
Detection of AF	0.15	1.0	0.26	0.85	0.14
Identification of group supraventricular extrasystoles (GSE)	0.42	0.88	0.57	0.61	0.55
Detection of AF + GSE	0.46	0.89	0.60	0.61	0.64
